# Amorphization and Nano-Crystallization of Ni-Nb Coating on GH3039 Alloys by High Current Pulsed Electron Beam

**DOI:** 10.3390/nano11020347

**Published:** 2021-02-01

**Authors:** Conglin Zhang, Xuesu Ji, Jiahong Wang, Lingfan Lu, Zirun Yang, Peng Lyu, Qingfeng Guan, Jie Cai

**Affiliations:** 1 School of Material Science and Engineering, Yancheng Institute of Technology, Yancheng 224051, China; conglin_zhang@126.com (C.Z.); 1437830211u@gmail.com (J.W.); llf000712@163.com (L.L.); 2 School of Materials Science and Engineering, Jiangsu University, Zhenjiang 212013, China; jxs97@hotmail.com (X.J.); lvp@ujs.edu.cn (P.L.); guanqf@ujs.edu.cn (Q.G.); 3 Institute of Advanced Manufacturing and Modern Equipment Technology, Jiangsu University, Zhenjiang 212013, China

**Keywords:** high current pulsed electron beam, Ni-Nb system, amorphous structures, mechanical property, corrosion resistance

## Abstract

In this paper, the Ni-Nb coatings were successfully prepared onto the GH3039 alloys by High current pulsed electron beam (HCPEB). The transmission electron microscopy (TEM) results confirmed that the Ni-Nb layer of 10-pulsed samples exhibited partial amorphization, which was consisted of γ-Ni particles, rod-like Ni_3_Nb particles and nano Ni_3_Nb with 30 nm in size. After 20-pulsed irradiation, the results show that only Ni_3_Nb clusters with around 3 nm in size were dispersed in fully amorphization layer. With increased pulse number to 30, the nano-particles embedded into the amorphous layer were grown up, the size of which was about 8 nm. The microstructure evolution during HCPEB irradiation was from the partial amorphous to fully amorphous and then to nano-crystallization. The 20-pulsed samples possessed the best hardness and corrosion resistance. The ultrafine clusters uniformly embedded into amorphous layer were main reason for improving properties.

## 1. Introduction

Amorphous structures have been prepared in the binary Ni-X systems, while Ni-Nb system is one of the best binary metallic glass forming systems due to their unique properties including excellent properties, high corrosion resistance and various magnetic properties [1,2,3]. Ni-Nb alloys exhibited a wide composition range (40–60%) to form amorphous structures. The best forming composition of the amorphous phase was Ni = 32% and Nb = 38% [4,5,6], which could be well confirmed by cluster formulas for stable liquids [7]. However, amorphous alloys possessed very low ductility as per-experiment study according to the reference [8]. Besides, it is hard for researchers to fabricate amorphous alloys to ensure its thermal stability that is another drawback. Under the heating condition, the free energy would be reduced through crystallization for the amorphous alloys. Nevertheless, several properties of amorphous alloys with the nanoscale crystal structure were found to be fundamentally different from, and often superior to, those of the conventional polycrystals and amorphous solids. For instance, nano-crystalline may enhance the strength, and ductility of materials and so on [9,10], which could remedy the above drawbacks. These nanocrystalline materials could have many potential applications, such as for surface protection and in nano-devices [11,12]. Pioneer explorations to study methods for nano-crystallization from amorphous state of Ni-Nb system was mainly included mechanical alloying [9,13,14], magnetron sputtering method [15,16] with the starting materials in the solid, powder or gaseous states. All of these methods have their inherent disadvantages for fabricating the amorphous alloys. The thin amorphous films were successfully prepared onto the substrate material through the sputtering method. However, there were the poor cohesion and adhesion between the film and substrate, limiting their potential biomedical and other applications. Mechanical alloying is only suitable for preparing powder materials. Therefore, searching for the suitable method to overcome these problems could be regarded as a better way.

The above problems could be solved by the formation of the alloying layer with amorphous structures in nanoscale size on the substrate. The alloying layer could be achieved by High current pulsed electron beam (HCPEB) irradiation as an innovative processing technique, which has been developed in recent years. Generally, this technique with many characteristics, such as short-duration pulse, high energy etc., was adopted to complete surface treatment. As inputting extremely intensive energy onto the topmost surface, the material surface was melted quickly and then solidified owing to the heat extraction towards the sub-surface. Under the effect of ultrafast melting and solidification, refined grains, even nano-grains and amorphous phase were formed which could be realized to promote the improvement of material properties [17,18,19]. HCPEB irradiation is an appropriate method for forming amorphous phase onto the surface layer. According to the reference [20], HCPEB irradiation promoted the formation of Ni-Nb amorphous layer onto the substrate to protect the substrate from corrosion. However, for the films/substrate system, the liquid-phase mutual diffusion impeded the amorphization process of melting layer and then resulted in the formation of nanocrystalline in the solidification process [21]. Meanwhile, supersaturated solid solution would be decomposed to form particles in nanoscale at the substrate [22,23]. Therefore, amorphous phase mixed nanostructures could be obtained by HCPEB technology, finally promoting a huge application of material. According to the previous literatures [17,18,19,20,21,22,23,24,25], investigation about amorphous mixed nanostructures and its evolution mechanism under HCPEB technology was few.

Therefore, in present research, the new Ni-Nb amorphous alloys have been prepared onto the GH3039 alloys by HCPEB irradiation. The aim of the present work was to investigate the microstructure evolution of Ni-Nb alloying layer for different number of pulses and their corresponding hardness and corrosion property.

## 2. Materials and Methods

**Figure 1:**
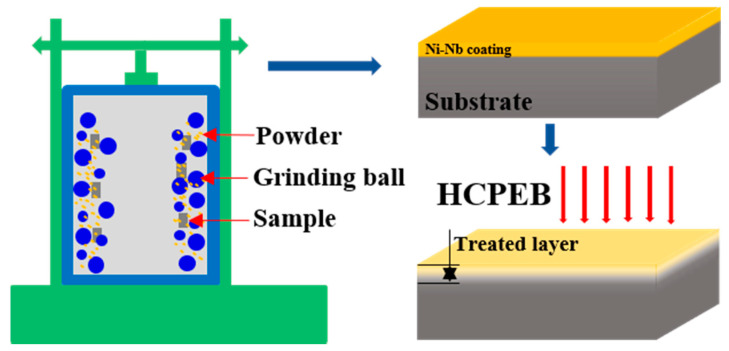
Schematic diagram of experimental process.

Ni-based GH3039 superalloy alloys with 10 × 10 × 5 mm^3^ in bulk-shaped were selected as substrate. The GH3039 superalloy was composed of C ≤ 0.08%, Mn = 0.40%, Mo = 1.80%, P = 0.02%, Al/Ti = 0.75%, Fe = 3.00%, Si = 0.80%, S = 0.012%, Cr = 22.00%, Nb = 1.30%, and Ni = balance. Before preparation of coating, all the samples were mechanically grounded with SiC papers (with 5.5 μm particle size), and then polished (with 1 μm diamond paste), finally cleaned. A mixture with chemical composition Ni_62_Nb_38_ was made by mixing commercially Ni (99.99% in purity) and Nb (98.0% in purity) powder. The mixed powder, GH3039 sample and a number of stainless-steel grinding balls were filled into the grinding vial. The prepared process was grinding duration of 3 h within argon gas at room temperature. As-fabricated single Ni-Nb coating was obtained onto the topmost of GH3039 alloy. After that, the as-fabricated coating was irradiated at room temperature using a HOPE-I type HCPEB device. The corresponding HCPEB parameters were the electron energy 27 keV, the beam diameter 60 mm, and the vacuum 5.0 × 10^−3^ Pa, energy density of 6 J/cm^2^, pulse duration of 1.5 μs and interval between each pulse of 10 s. The fabricated and treated process was schematically exhibited in Figure 1.

The X-ray diffraction (XRD, Rigaku, Tokyo, Japan) was carried out on the CuKa radiation in a D/max-2500/pc X-ray diffractometer to verify phase structures. The surface morphology of Ni-Nb coating was observed by using FEI Nova Nano 450 scanning electron microscope (SEM, FEI Company, Hillsborough, OR, USA) with an Inca energy 350 energy-dispersive spectrometer (EDS) at 15 kV acceleration voltage. The microstructure was carried out on the JEM-2100 transmission electron microscopy (TEM, JEOL, Tokyo, Japan) in the combination with selected area electron diffraction (SAED). The mechanical property of Ni-Nb coating was examined by an HVS-1000 microhardness measurement device (Shanghai fuley measuring equipment Co., Ltd., Shanghai, China) with a load of 0.245 N (25 g) applied for 15 s. The corrosion property of the Ni-Nb coating with 1 cm^2^ in area was tested with a CHI760C electrochemical workstation (Chinstruments Co., Ltd., Shanghai, China) in the 3.5 wt% NaCl (0.6 M) water solution. The cyclic polarization was done at a sweep rate of 0.333 mV/s. A saturated calomel electrode, platinum and Ni-Nb coating were served as the reference electrode, the counter electrode and the work electrode, respectively.

## 3. Results

**Figure 2:**
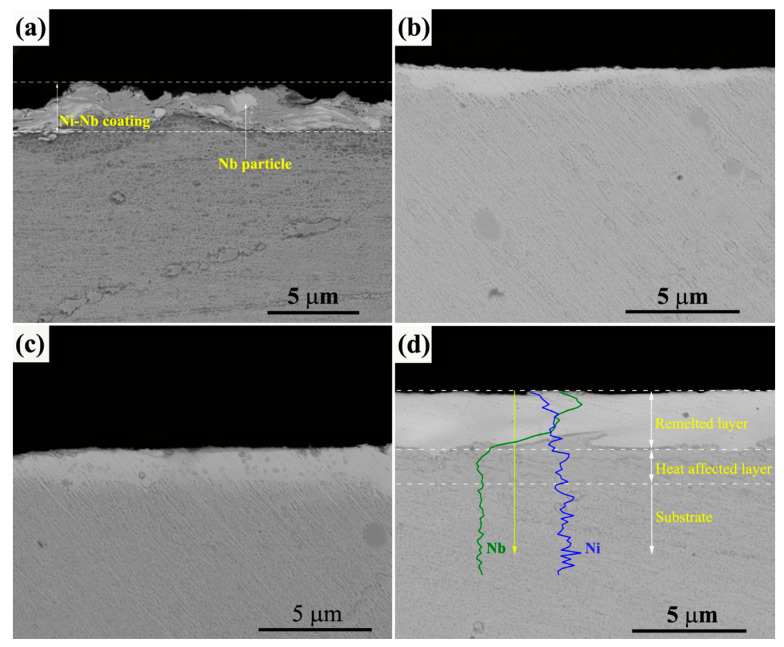
The cross-section BSE images of initial and irradiated Ni-Nb coating. (**a**) Initial samples; (**b**) 10 pulses; (**c**) 20 pulses; (**d**) 30 pulses.

Figure 2 gives the typical cross-section BSE images of initial and irradiated Ni-Nb coating. As shown in Figure 2a, the Ni-Nb coating with an average thickness of 1.9 μm was successfully prepared on the surface of GH3039 substrate. The as-fabricated coatings with tiny pore-structures were relatively dense. Besides, Nb particles were clearly observed, indicating that the Ni and Nb elements were no uniformly distributed in the coating. The probable reason was the short milling time and high energy collision to result into insufficient energy input during the ball milling. In addition, cold welding occurred due to the short milling time. The clear interface was formed between the as fabricating a coating and the substrate. After 10-pulsed irradiation, the top layer was re-melted and the thickness of which was only about 0.64 μm as compared to the initial coating, as shown in Figure 2b. With an increment of the pulses number, the thickness of HCPEB alloyed layer was increased from 1.5 μm for 20-pulsed sample (Figure 2c) to about 2.5 μm for the 30-pulsed sample. One can see that the morphology of all the irradiated samples exhibited a relatively white layer from the contrast. There was excellent metallurgical bonding that increased the coating density and reduced the interface defect for the irradiated samples. Especially, for the 30-pulsed samples, the alloying layer can be clearly divided into three layers: re-melted layer, heat affected zone and the substrate. From the line scanning result inserted in Figure 2d, there was obvious Nb-enriched element at the beginning of the line scanning, which was corresponded to the re-melted layer. Besides, it could be seen that the Nb element was present in the heat affected layer that was resulted from the dissolution of the Nb. Therefore, it is considered that the Ni-Nb alloying layer was formed onto the substrate after HCPEB irradiation.

**Figure 3:**
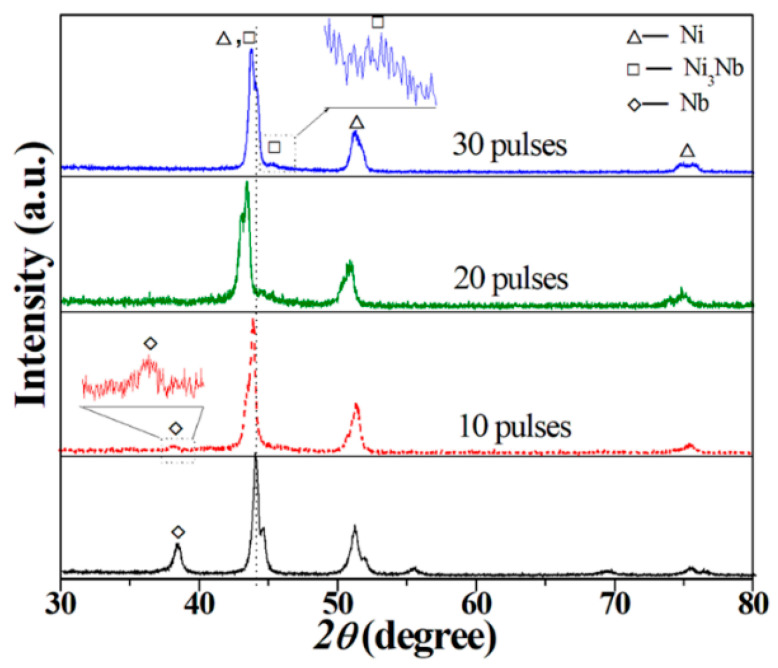
XRD pattern of the initial Ni-Nb coating and after HCPEB irradiation.

Figure 3 depicts a typical XRD pattern of the initial Ni-Nb coating and after HCPEB irradiation. The reasons of phase transformation were allowed to make several assumptions due to surface treatment. Firstly, the diffractograms exhibit some peaks of the Ni, Nb and Ni_3_Nb phase in the irradiated layer. Noticeably, Nb peak at 2ϴ = 38° with low intensity still existed in the XRD pattern after 10-pulsed irradiation. With pulses increased to 20, the Nb peak was disappeared, which suggests the formation of Ni_3_Nb or Ni(Nb) solid solution. Additionally, Ni diffraction peaks have shifted to low angle after 20-pulsed irradiation comparing to the initial sample, meaning that the Ni lattices were expanded, which indicates that Ni(Nb) solid solution was formed. For the 30-pulsed samples, a minor shift of Ni peaks to high angle occurred. The possible reason was that the Nb atoms were decomposed from the Ni(Nb) solid solution to form Ni_3_Nb phase. Especially in the pattern of 30-pulsed Ni-Nb samples, the δ-Ni_3_Nb phase was clearly identified. Besides, the broader diffractograms peaks of Ni were resulted from the formation of amorphous phase due to the distorted lattice. Certainly, all of the grain refining, dislocation density increasing and micro stress accumulating resulted into broader peaks. The specific reason needs further detailed characterization by TEM to elaborate in the following.

**Figure 4:**
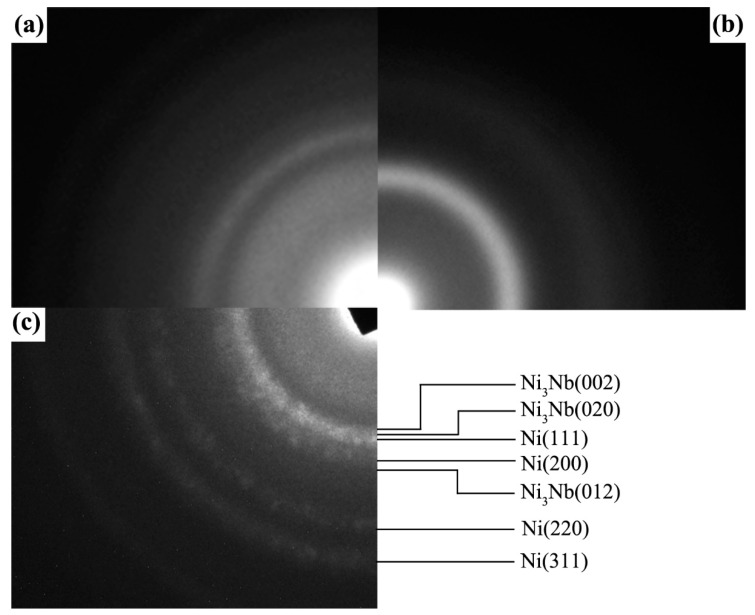
SAED patterns of local area for Ni-Nb coating with different number of pulses. (**a**) 10 pulses; (**b**) 20 pulses; (**c**) 30 pulses.

Figure 4 shows the SAED patterns of local area for Ni-Nb coating with different number of pulses. From Figure 4a–c, it could be considered that an amorphous phase was formed in the irradiated samples, but the atomic range order of which was different. After 10-pulsed irradiation, the SAED pattern gives three broader rings with low diffusion (Figure 4a), suggesting the atom ordered arrangement of crystal structure still existed in the characterized local area. Namely, the degree of amorphization was low. With the pulse number increased to 20, only two diffraction rings were observed in the SAED pattern (Figure 4b), indicating the ordered arrangement of only the nearest neighbor and next nearest neighbor and the disordered arrangement of the third adjacent atoms. Therefore, it could decide that these structures have been completely amorphous. However, after HCPEB irradiation with 30 pulses, the SAED pattern in Figure 4c exhibits that the amorphous halo coexists with several weak diffraction speckles, suggesting that the nano-particles were separated out within the measured area. Therefore, the structural transformation in the Ni-Nb layer could be described as from partial amorphous to fully amorphous and then to nano-crystalline from the perspective of the SAED patterns due to HCPEB irradiation.

**Figure 5:**
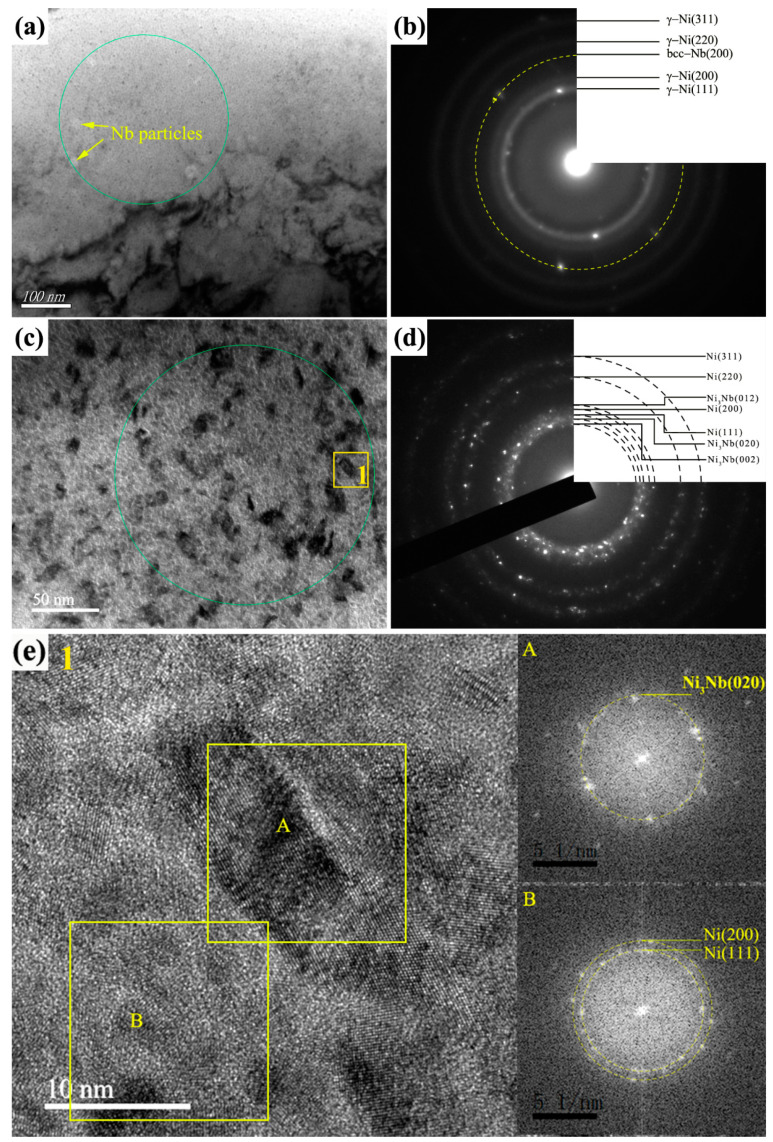
TEM images and SAED patterns of the 10-pulsed Ni-Nb coating. (**a**) The Ni particles and Ni_3_Nb particles; (**b**) the SAED pattern of (**a**); (**c**) higher magnification image of the Ni and Ni_3_Nb particles; (**d**) The SAED pattern of (**c**); (**e**) The HRTEM image of region 1.

In addition, a large number of nano black particles and blocky-shaped structures with about 30 nm in size were formed in some area, as shown in Figure 5c, the SAED (taken from the green circle in the Figure 5c) of which demonstrated that the Ni phase was coexisted with the Ni_3_Nb phase (Figure 5d). For further examining the morphology of Ni and Ni_3_Nb phase, the HRTEM image (magnification of the region 1) and the Fast-flourier transformation (FFT) pattern of enlarged image was exhibited in the Figure 5e. For the A zone, the d value of the measure area correspond well with d(020) = 0.212 nm for the δ-Ni_3_Nb, and for the B zone, d(111) = 0.203 nm and d(200) = 0.176 nm for the FCC Ni phase. Therefore, it could be concluded that nano black particles were the mixture of γ−Ni particles and Ni_3_Nb particles with the blocky-shaped structures.

**Figure 6:**
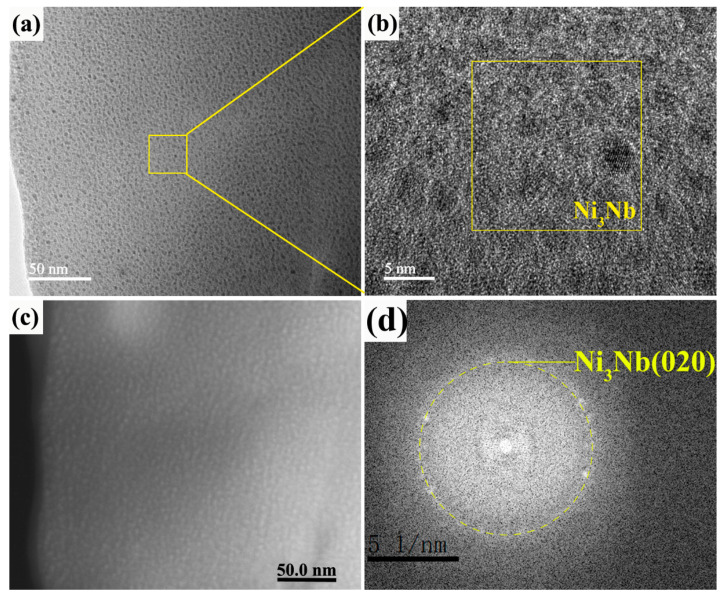
The TEM images, HADDF image and FFT pattern of the 20-pulsed Ni-Nb layer. (**a**) The amorphous phase; (**b**) The HRTEM image of local area in (**a**); (**c**) The HADDF image of amorphous matrix; (**d**) The corresponding SAED pattern of (**b**).

Figure 6a,b give the bright field and HRTEM images of 20-pulsed Ni-Nb layer. Although the SAED of 20 pulsed samples indicates that the matrix was composed of the amorphous phase (Figure 4b), the very fine clusters crystals with about 3 nm in size were still formed in the amorphous layer on the topmost matrix, as shown in Figure 6a. From the HRTEM image, the individual nanoparticles were separated by envelopes of the amorphous matrix. The FFT pattern taken from the Figure 6b indicates that the d value was equal with 0.2023 nm, which was Ni or Ni_3_Nb clusters. Figure 6c,d exhibit the images of 20-pulsed Ni-Nb coating through high angle angular field (HAADF) and the corresponding elements distribution. It could be concluded from the results of HAADF image that the element with large atomic number segregated at the spherical microstructures, as shown in Figure 6c. From Figure 6d, the spectrum analysis result indicates that the spherical microstructure was consisted of the Nb element dispersed in the Ni matrix. Therefore, combined of the HRTEM image, these results confirmed that the spherical microstructures were Ni_3_Nb particles. In addition, different from the 10-pulsed samples, no large size Nb particles were observed in the 20-pulsed layer, which suggests that Nb was completely dissolved into the Ni amorphous matrix.

**Figure 7:**
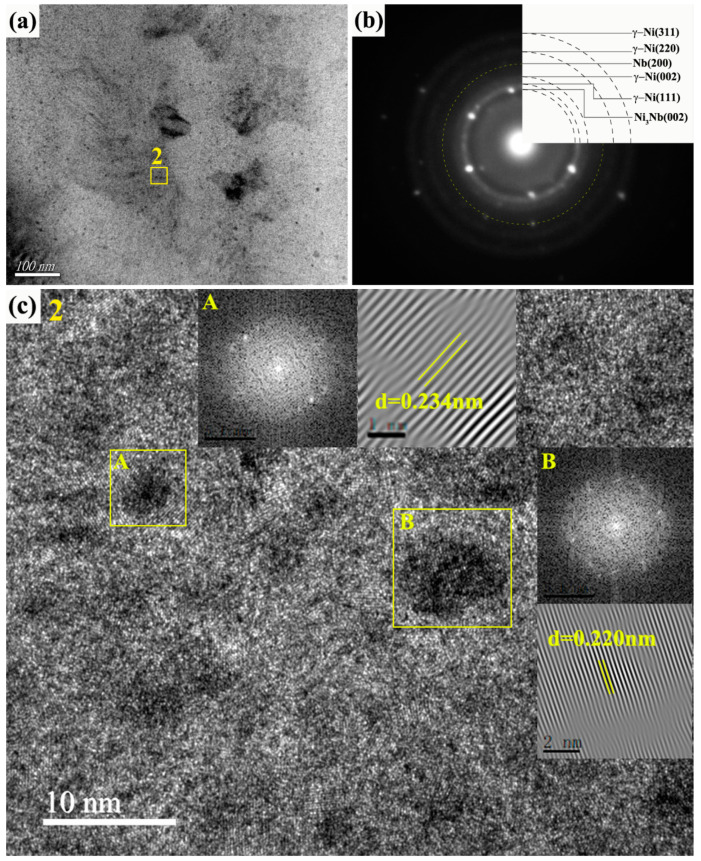
The TEM image, SEAD pattern, and HRTEM image of 30-pulsed Ni-Nb layer. (**a**) The TEM bright field image of Ni-Nb layer; (**b**) The SEAD pattern of (**a**); (**c**) The HRTEM image of region 2 in (**a**).

After 30-pulsed irradiations, a little change in microstructure took place in the alloying layer. There was also consisted of amorphous matrix and the nano particles embedded in it, but the size of which increased to about ~8 nm, as shown in Figure 7a. The SAED pattern exhibits the diffuse ring along with several pairs of diffraction spots that confirmed the presence of a homogeneous amorphous structure with amounts of finely-dispersed nanocrystalline Ni_3_Nb. Noticeably, faint diffraction speckles were observed in Figure 7b, which suggested the formation of ultrafine Nb particles that were the product of precipitation from the γ-Ni. Besides, the HRTEM image (magnification of the region 2) was used to demonstrate the presence of Ni_3_Nb and Nb particles, as shown in Figure 7c. The FFT and inverse FFT patterns corresponding to the A zone was indexed as the Nb phase, and that of the B zone was confirmed as Ni_3_Nb phase. Therefore, it can be concluded that the alloying layer was consisted of nano Νb and Ni_3_Nb particles that were uniformly dispersed into the Ni-Nb amorphous matrix after 30-pulsed irradiation.

**Figure 8:**
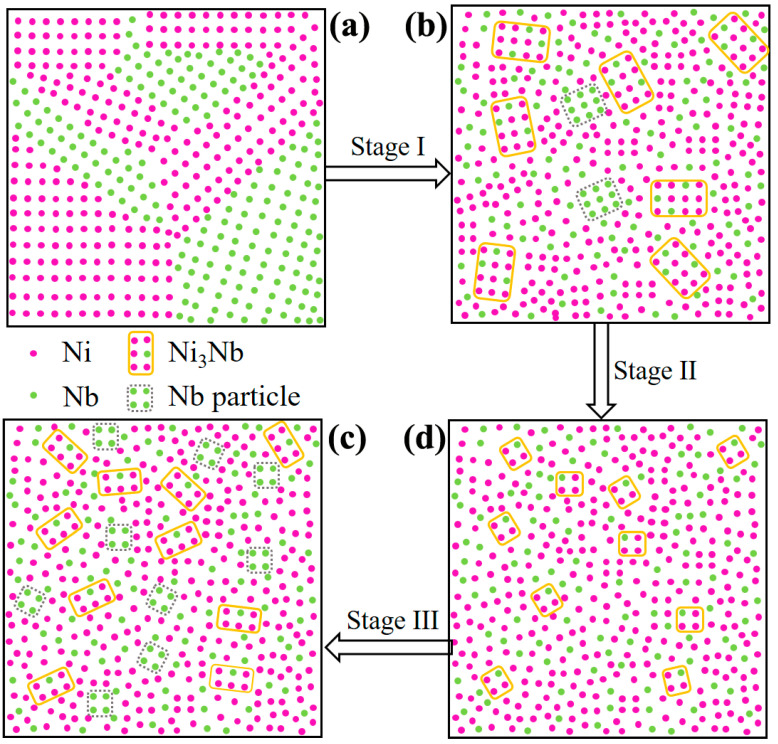
A schematic microstructure evolution for HCPEB irradiation. (**a**) Initial stage; (**b**) Partial amorphous; (**c**) Fully amorphous; (**d**) Nano-crystallization.

Based on the experimental results and observations at different stages of HCPEB irradiation, a microstructure evolution is proposed for HCPEB irradiation, as shown by a schematic in Figure 8. Under as-milled condition, all the atoms are uniformly arranged due to short milled time, as shown in Figure 8a. After 10-pulsed irradiation, the amorphous phase was formed in the Ni-Nb layer, which could be got from Figure 4a. The rapid cooling is responsible for the amorphization of the Ni_60_Nb_40_ coatings due to HCPEB irradiation. However, from Figure 5, there were still the Ni, Nb particles and intermetallic Ni_3_Nb with large size dispersed in the amorphous Ni-Nb layer (Figure 8b). Hence, the stage I was identified as partial amorphization stage. With increasing the pulses number to 20, the residual Nb particles were continuously dissolved into the amorphous matrix to aggravate the amorphization. Yet, it is inevitable that Ni_3_Nb clusters were formed and uniformly distributed in the amorphous Ni-Nb layer (see Figure 5). Therefore, the stage II could be called fully amorphization process comparing to the stage I (Figure 8c). The more energy inputting is responsible for the fully amorphous of the Ni_62_Nb_38_ layer, which have reduced the diffusivity and retarded rearrangement of all atoms after proceeding uniform mixing of Ni and Nb element. Meanwhile, the number of nucleation sites could be increased during 20-pulsed irradiation, restraining the coarsening of Ni_3_Nb clusters at this stage. In addition, the atoms with the difference in the atomic size changed in the order Nb (0.143 nm) > Ni (0.125 nm), together with the highly negative mixing enthalpy in the Ni-Nb (−30 kJ/mol) [26,27]. All of the above factors could make it hard to arrange the Ni and Nb element on a long-range scale under the ultra-rapid cooling rates condition during the HCPEB irradiation. Therefore, during the stage II of HCPEB irradiation, the Ni_3_Nb clusters have grown to only ~3 nm in size, as shown in Figure 6a. Actually, it indicates that the onset of primary crystallization has begun in the process of fully amorphization.

On base of the stage II, the size of nano-Ni_3_Nb clusters coarsened up to 8 nm in size with increasing pulses number. Meanwhile, a large number of Nb nano-particles has already been formed during this stage. This strongly supported that the chemical fluctuation to break the Ni/Nb component ratio (Ni:Nb = 62:38) in the local area. It is considered that the growth of Ni_3_Nb particles is due to composition fluctuation existed in the liquid metal [28]. Owing to the ultra-short time (1.5 μs) of HCPEB irradiation, it is hard to grow up for the Nb and Ni_3_Nb nano-particles, as shown in Figure 7c. Therefore, nano-crystallization (stage III) from a fully amorphous state during HCPEB irradiation enforced crystallization of locally enriched regions under rapid cooling rates (Figure 8d). In addition, with increasing thickness of Ni-Nb coating, Ni element from the GH3039 matrix has dissolved into the Ni_62_Nb_38_ alloying layer also to break the Ni/Nb component ratio (the best forming amorphous ratio of Ni to Nb is 62:38), which makes it to deviate proper component of amorphous phase. Finally, the formation of nano-crystalline inevitably took place to decrease the content of amorphous phase for 30-pulsed Ni-Nb layer. Therefore, it could be concluded that the microstructure evolution proposed here was partial amorphous to fully amorphous and then to nano-crystalline during HCPEB irradiation.

**Figure 9:**
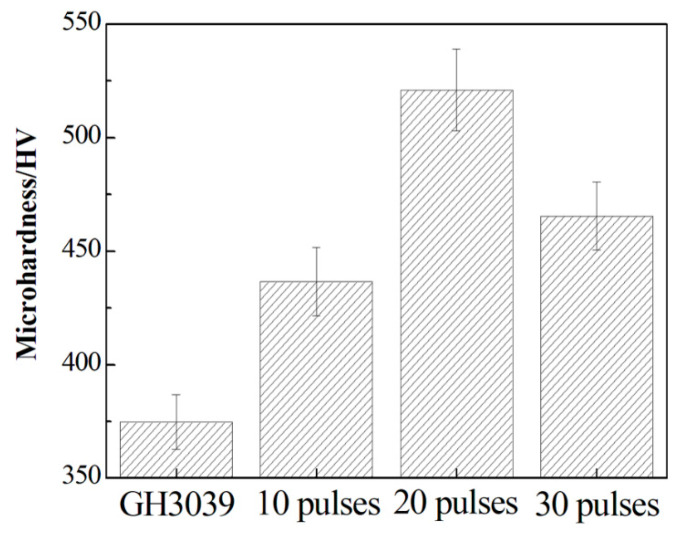
The hardness values for the Ni-Nb layer with the different number of pulses.

To investigate the mechanical property of Ni-Nb coating, the microhardness testing was performed. Figure 9 gives the hardness values for the Ni-Nb coating with the different pulses. For the initial sample, the hardness was about 375 HV; while the hardness value was 425, 520 and 458 HV after HCPEB irradiation, corresponding to 10, 20 and 30 pulses, respectively. It can be seen that the microhardness values had an increasing trend with increasing up to 20 pulses and then dropped slightly after 30 pulses. On the whole, the hardness was dramatically improved after HCPEB irradiation. The mainly reason for increasing hardness was the formation of mixing amorphous and nanocrystalline structures. Vickers hardness suggested the bonding strengthen inside alloys, and was referred to one of intrinsic parameters to characterize the mechanical properties of alloys [29]. The number of metallic bonding between adjacent atoms was increased in Ni-Nb layer to result in the increasing the number of atoms connected by metallic bonding. Then the higher hardness value could be obtained due to the presence of no directed bonding in the binary Ni-Nb amorphous layer. Therefore, the fully amorphized structures for 20-pulsed samples together with the uniformly distributed Ni_3_Nb particles resulted in the increasing strengthen, enabling the Ni-Nb layer hardening. It was understandable that the decreasing hardness of 30-pulsed samples was owing to the precipitation of Nb nano-particles, damaging reduction of amorphous phase strengthen. Meanwhile, the coarsening of dispersed Ni_3_Nb nanocrystals occurred during HCPEB irradiation. So, these factors were making hardness for the 30-pulsed samples to decrease. In conclusion, samples hardening was mainly attributed to the metal-metal interatomic bonding strengthen, nanocrystalline strengthen and so on [23,30].

**Figure 10:**
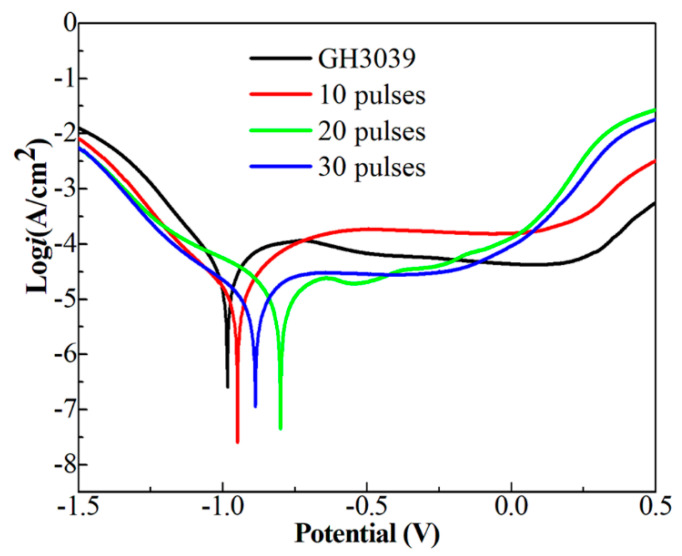
The polarization curves of the Ni-Nb coating before and after HCPEB irradiation.

Figure 10 gives the polarization curves of the Ni-Nb coating before and after HCPEB irradiation and the corresponding corrosion current ((i_corr_) and corrosion potential (E_corr_) were calculated and tabulated in Table 1. From Figure 10, it can be observed that the polarization curve is divided into four parts: active region, passive transition zone, passive stabilization zone and active passive zone [31]. At the early stage, there were the crystal defects seen in the many HCPEB treated metals [20,25] with energy concentration inside microstructure at the topmost layer, which was easy to be corroded preferentially, forming the active region. Then hydrolysis of corrosion products promoted the formation of dense passivation film, resulting in a stable passivation area. The E_corr_ and the I_corr_ of the initial samples were −0.990 V and 28.90 μA·cm^−2^, respectively, as shown in Table 1. After HCPEB irradiation, the E_corr_ and I_corr_ of specimens were changed apparently. The Ecorr of 10- and 20-pulsed samples was increased to −0.904 V and −0.799 V and I_corr_ decreased to 10.2 μA/cm^2^ and 6.98 μA/cm^2^, respectively. As increasing the pulses number up to 30, the I_corr_ increased to 7.83 μA/cm^2^ and the E_corr_ decreased to −0.887 V. Therefore, a drastic enhancement of corrosion resistance was obtained for the Ni-Nb layer with 20 pulses compared to that of other samples.

Generally, the corrosion resistance of amorphous alloys was superior to the crystalline metal material. However, the Ni-Nb alloys only with amorphous structure have a characterization of the cracks in the reference [20]. It destroyed the surface integrity and aggravated the damage of the surface of the Ni-Nb alloy. However, in present paper, these cracks could be released or eliminated under the circumstance of the amorphous layer with the nanoscale crystal structure, which improved the toughness and plasticity [9,10]. Therefore, the enhancement in corrosion resistance of Ni-Nb layer was naturally connected with the formation of nanocrystalline and amorphous phase. The 20-pulsed samples have the best corrosion resistance in the test. In addition, the corrosion resistance for the different pulses samples also has been affected by the formation of crystal defects, deformed structures, solid solution precipitation and intermetallic compounds as reported in the references [32,33], which will be confirmed by further characterization in the future.

## 4. Conclusions

The main findings are summarized as follows:

1. The dense Ni-Nb coating with ~1.9 μm in thickness was fabricated onto GH3039 alloys, in which the distribution of Ni and Nb elements was not uniform. After HCPEB irradiation, the amorphous layer was fabricated onto the substrate, the thickness of which was increased with increasing number of pulses. The layer could be divided into re-melted layer and heat affected layer that was about 2.5 μm in thickness for the 30-pulsed sample.

2. After 10-pulsed irradiation, the whiter Nb particles and ultrafine black particles corresponding to γ-Ni particles and Ni_3_Nb particles with 30 nm in size existed in the alloying layer. There were also δ−Ni_3_Nb particles characterized by rod-like structure distributed in the amorphous matrix.

3. In 20-pulsed samples, Nb particles and Ni_3_Nb particles in large size were dissolved to form Ni_3_Nb in nanometer uniformly distributed into the amorphous layer. Compared to the 20-pulsed sample, the size of nano-particles embedded into the amorphous layer was increased after 30-pulsed irradiation, reached to about ~8 nm.

4. SAED patterns of the Ni-Nb layer changed from three broader rings with low diffusion to only two diffraction rings and then to amorphous halo coexisted with several weak diffraction speckles. Combined with the microstructure, the structure evolution during HCPEB irradiation could be described as from the partial amorphous to fully amorphous and then to nano-crystallization.

5. The hardness and corrosion resistance of Ni-Nb amorphous layer were significantly improved after HCPEB irradiation. The 20-pulsed sample obtained the best results. The main factor contributing to such improvement was the formation of Ni_3_Nb particles in nano size uniformly distributed in the amorphous layer.

## Figures and Tables

**Table 1:** Icorr and Ecorr of the Ni-Nb coating before and after HCPEB irradiation.

Samples	E_corr_ (V)	I_corr_ (μA·cm^−2^)
GH3039	−0.990	28.90
10 pulses	−0.940	10.20
20 pulses	−0.799	6.98
30 pulses	−0.887	7.83
